# Factors associated with bone metabolism in patients with cervical ossification of the posterior longitudinal ligament accompanied with diffuse idiopathic skeletal hyperostosis

**DOI:** 10.1051/sicotj/2017061

**Published:** 2018-03-16

**Authors:** Shinji Horie, Yasunobu Sawaji, Kenji Endo, Hidekazu Suzuki, Yuji Matsuoka, Hirosuke Nishimura, Takeshi Seki, Kengo Yamamoto

**Affiliations:** Department of Orthopedic Surgery, Tokyo Medical University, 6-7-1 Nishishinjuku, Shinjuku-ku, Tokyo 160-0023 Japan

**Keywords:** Diffuse idiopathic skeletal hyperostosis, Cervical ossification of the posterior longitudinal ligament, Bone metabolism factor, Parathyroid hormone

## Abstract

*Introduction*: Diffuse idiopathic skeletal hyperostosis (DISH) and ossification of the posterior longitudinal ligament (OPLL) are both characterized as ossification in paravertebral ligaments and sometimes present simultaneously, however, the bone metabolism in patients with cervical OPLL accompanying/not accompanying DISH has not well been studied. Thus, a retrospective analysis was performed to understand any differences in bone metabolism in these patients.

*Methods*: Male patients who underwent surgery for OPLL were divided into two groups based on the presence or absence of DISH (OD and O group, respectively). Patients with cervical spondylosis comprised the control group (CS group). Bone mineral density (BMD) and bone metabolism factors were compared among the groups.

*Results*: The OD and O groups had significantly higher body mass indexes (BMIs) than did the CS group. Morphologically, the number of continuous type of OPLL was high in the OD group whereas that of segmental type was higher in the O group. The OD and O group had greater BMD than the CS group. Both TRACP-5b and P1NP were tended to be lower in the OD group whereas Ca and P concentrations were similar level among the groups. Intact parathyroid hormone in OD group was significantly higher than CS group.

*Discussion*: Patients with OPLL accompanying DISH had significantly higher BMD whereas they tend to be lower in bone turnover. Significantly higher i-PTH levels was found in the OD group and would be the characteristic blood marker, but further research on the relationship between DISH and PTH was necessary.

## Introduction

Ossification of the posterior longitudinal ligament (OPLL) generally occurs in the cervical spine, and diffuse idiopathic skeletal hyperostosis (DISH) commonly occurs in the thoracic spine; thus, these two diseases have been considered as different disease states. However, Fujimori et al. reported that the concomitant rate of cervical OPLL accompanying DISH was 36% [1] and Resnick et al. showed 50% of cases of DISH were concomitant with OPLL [2]. Therefore, OPLL has been recognized as one of the clinical features of DISH in recent years.

DISH is a systemic disease involving ossification of the ligaments (mostly involving the spine) that was first reported by Resnick et al. [3] in 1975. Previous epidemiologic studies have shown that the prevalence of DISH in Japanese and other Asians ranged from 5.4% to 38.7% in men and from 0.8% to 13.9% in women, and the mean age of these patients ranged from 65.3 to 66.7 years [4–6]. Other studies that investigated multiple types of ossification of the ligaments, including OPLL, ossification of ligamentum flavum, ossification of anterior longitudinal ligament, DISH, and ossification of nuchal ligament, reported that there were more patients with multiple combined types of ossification than there were those with only one type [1]. Additionally, both patients with one type and those with multiple types of ossification had significantly greater bone mineral density (BMD) than did those with no ossification [7–9].

It is well known that, in patients with cervical OPLL, cervical spinal cord injury may occur due to even a minor trauma [10]. Some studies have reported that recent trends in population ageing are also associated with the incidences of severe spinal fractures and spinal cord injuries in patients with DISH [11–13]. Thus, the presence of ossification in spine at multiple levels are of importance clinically, however, few studies have examined the characteristics of bone metabolism in patients with cervical OPLL accompanying DISH [14].

Therefore, a retrospective analysis was performed to understand any differences in bone metabolism by investigating patients' demographics, type of OPLL and bone turnover marks and compared these parameters among patients with OPLL alone, OPLL accompanying DISH and patients without ossification.

## Materials and methods

There were 49 male patients (mean age, 59.6 years) who underwent surgery for C-OPLL in our institution between 2010 and 2016. Plain radiography and whole-spine computed tomography (CT) were performed, and the presence or absence of DISH was evaluated in accordance with the definition by Resnick [3]. According to the presence or absence of DISH, patients were divided into two groups: the OPLL accompanying DISH (OD) group and the OPLL (O) group. Patients who underwent surgery for cervical spondylosis (CS) during the same period (26 age-matched male patients; mean age, 56.8 years) were included in the CS group (control group). Based on the CT image, we classified each OPLL case into four types according to the OPLL classification reported by Tsuyama [15]. Among the 49 patients with C-OPLL, 25 (51%) were included in the OD group and 24 (49%) in the O group (Table 1). The following items were investigated in the three groups: BMD of the femoral neck and the lumbar spine using dual-energy X-ray absorptiometry, tartrate-resistant acid phosphatase 5b (TRACP-5b) as a bone resorption marker, procollagen type 1 nitrogenous propeptides (P1NP) as a bone formation marker, and intact parathyroid hormone (i-PTH), blood calcium (Ca), and blood phosphorus (P) as other factors associated with bone metabolism. Patients who had renal dysfunction were excluded because these markers are known to be affected [16].

**Table 1:**
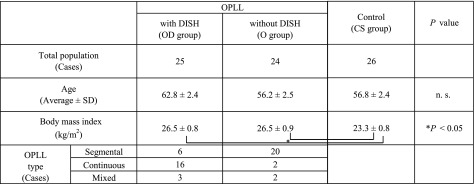
The number of patients, mean age and body mass index (mean ± standard deviation), and ossification of the posterior longitudinal ligament classification type for each group.

JMP^®^ 11.0 (SAS Institute Inc., Cary, NC, USA) was used for statistical data analysis. The statistical tests employed were the chi-squared test for classification by types of C-OPLL and the F-test in the one-way analysis of variance (ANOVA) for multiple comparisons of other variables.

## Results

The OD and O groups had significantly higher body mass indexes (BMIs) than did the CS group; however, no significant difference in BMI was noted between the OD and O groups. Regarding the type classification of OPLL, the number of continuous type was found to be significantly higher in the OD group than in the O group, whereas the number of segmental type was significantly high in the O group (Table 1).

The OD group had significantly greater BMD in both the femoral neck and lumbar spine than the O and CS groups did. Both bone resorption marker, TRACP-5b and bone formation markers, P1NP were tended to be lower in the OD group compared with that in the O and CS groups, although they did not reach statistical significance. No significant differences were noted for blood Ca or P among the three groups. Interestingly, i-PTH concentration in the OD group was higher than that in the O group and it was statistically significantly higher than that in the CS group (Table 2).

**Table 2:**
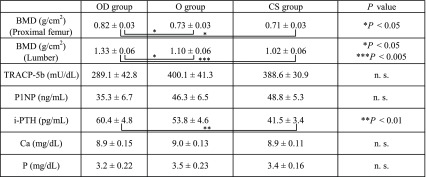
Bone mineral density and factors associated with bone turnover (mean ± standard deviation) for each.

## Discussion

Fujimori et al. [1] conducted a study in 1500 Japanese subjects and reported that 36% of the subjects with OPLL also had DISH. The concomitance rate in the current study was somewhat higher (51%), presumably the subjects in our study were symptomatic and advanced stages of C-OPLL for which surgery was indicated. Tauchi et al. [17] investigated the type of OPLL with or without DISH and found that all of the patients who had both OPLL and DISH had continuous and mixed type, whereas 73% of patients with OPLL without DISH had segmental type. Together with our results (Table 1), patients with OPLL accompanying DISH tend to be osteogenic at other vertebral ligaments and to have continuous type of OPLL.

Consistent with our result that patients with OPLL accompanying DISH have significantly higher lumbar spine and femoral neck BMD than control subjects did was reported previously [9]. Furthermore, in our study showed that the OD group had greater BMD than the O group did, which would suggest that the OD group is more osteogenic than O group. Previous study [18] showed high BMIs after age 20 and diabetes mellitus are independent factor for OPLL.

Regarding bone turnover markers, Kashii et al. [19] reported that patients with OPLL had significantly lower TRACP-5b and P1NP levels than patients without OPLL did. In our study, both TRACP-5b and P1NP levels in the O and OD groups also tended to be lower than those in the CS group, although it did not reach statistical significant differences, suggesting that systemic bone turnover in patients with OPLL accompanying DISH tended to be decreased.

i-PTH is a hormone whose secretion is facilitated or suppressed according to the activity of vitamin D in the body. Okazaki et al. [20] reported that hypoparathyroidism was found in a high proportion of patients with OPLL. Kashii et al. [19] reported that i-PTH levels were high in OPLL patients and positively correlated with sclerostin levels in the blood. Oleson et al. [21] reported that high blood levels of PTH were found in 75% of patients with spinal cord injuries with ectopic ossification and that low levels of vitamin D were found in 92% of these patients. Our study showed that the OD group had significantly higher i-PTH levels than the CS group did; accordingly, i-PTH was considered likely to be involved in increased bone growth in the OD group. PTH could either act as catabolic or anabolic in the bone homeostasis depending on periodicity and length [22]. PTH is able to exert anabolic effects upon intermittent administration; clinically used as osteo-anabolic therapy for the treatment of osteoporosis. A basic experiment comparing the phenotype of ligament cells from normal subject or patients with OPLL revealed that the cells from OPLL showed the osteoblast-like phenotype in response to PTH, but cells from ligament without ossification did not show any osteoblastic properties, [23] suggesting that ligament cells of OPLL are responsive to PTH associating with bone formation. However, it remains unknown the diurnal variation and level of the local PTH in patients with OPLL with or without DISH, further investigations need to be done. It should be noted that because the PTH level is influenced in patients with renal dysfunction and post-menopausal osteoporosis [16], we excluded these patients in this study.The present study had the following two limitations: (1) The sample size was relatively small because all of the subjects were patients with severe OPLL for which surgery was indicated; thus, if patients with mild OPLL were included, the study might have shown different results; and (2) Patients with DISH alone were not compared with those with OPLL alone (O group) in this study; thus, bone metabolism in the presence of OPLL versus DISH could not be compared. Further studies must be conducted in patients with asymptomatic OPLL and DISH. However, as there have been no studies presenting evidence for increased i-PTH in patients with OPLL complicated by DISH for which surgery is indicated, we believe that this study makes a significant contribution to clarifying the endocrinologic status associated with ossification of the ligaments.

## Conclusions

Patients with OPLL accompanying DISH had significantly higher BMD whereas they tend to be lower in bone turnover. Significantly higher i-PTH levels was found in the OD group and would be the characteristic blood marker, but further research on the relationship between DISH and PTH was necessary.

## Ethical approval

All procedures performed in studies involving human participants were in accordance with the ethical standards of the institutional and/or national research committee and with the 1964 Helsinki declaration and its later amendments or comparable ethical standards. For this type of study, formal consent is not required.

## Conflict of Interest

The authors declare that they have no conflicts of interest in relation to this article.
